# Biological gene extraction path based on knowledge graph and natural language processing

**DOI:** 10.3389/fgene.2022.1086379

**Published:** 2023-01-13

**Authors:** Canlin Zhang, Xiaopei Cao

**Affiliations:** ^1^ Sorenson Communications, Salt Lake City, UT, United States; ^2^ College of Creative Culture and Communication, Zhejiang Normal University, Jinhua, Zhejiang, China

**Keywords:** biological gene extraction, knowledge graph, natural language processing, path research, biological gene

## Abstract

The continuous progress of society and the vigorous development of science and technology have brought people the dawn of maintaining health and preventing and controlling diseases. At the same time, with the update and iteration of bioinformatics technology, the current biological gene research has also undergone revolutionary changes. However, a long-standing problem in genetic research has always plagued researchers, that is, how to find the most needed sample genes from a large number of sample genes, so as to reduce unnecessary research and reduce research costs. By studying the extraction path of biological genes, it can help researchers to extract the most valuable research genes and avoid wasting time and energy. In order to solve the above problems, this paper used the Bhattacharyya distance index and the Gini index to screen the sample genes when extracting the characteristic genes of breast cancer. In the selected 49 public genes, 6 principal components were extracted by principal component analysis (PCA), and finally the experimental results were tested. It was found that when the optimal number of characteristic genes was selected as 5, the recognition rate of genes reached the highest 90.31%, which met the experimental requirements. In addition, the experiment also proved that the characteristic gene extraction method designed in this paper had a removal rate of 99.75% of redundant genes, which can greatly reduce the time and money cost of research.

## 1 Introduction

In a rapidly developing world, various diseases are increasingly threatening people’s healthy life. Many of these diseases can be resisted through physical defense, but many diseases come from human genes. How to study human-related diseases and find solutions for corresponding diseases is a difficult problem that cannot be ignored in current life sciences. Each genetic disease has its own characteristics, and the ever-changing gene expression is the key to preventing medical staff from curing such diseases. Coincidentally, how to extract the most needed genes when studying disease genes is also the key for researchers to solve the problem of genetic diseases. From the above description, the importance of biological gene extraction pathway research can be found. In order to solve this problem, this paper selects the theory-assisted research of knowledge graph and natural language processing.

This paper adopts the knowledge graph and natural language processing theory for the research on the extraction path of biological genes, the purpose of which is to apply the technology of knowledge extraction of knowledge graph to the extraction of biological genes. In this way, the extraction of genes in clinical medicine and biological research can be better achieved, thereby further promoting the development of life sciences. The Bhattacharyya distance index and Gini index used in this paper have a good screening effect on massive research samples, and the PCA method can also further realize the extraction of characteristic genes. The innovation of this paper includes the following aspects: 1) It is not limited to the activity research of specific genes, but seeks to reduce the research cost and optimize the research process of similar research from the methodological level. 2) The combination of knowledge graph and gene extraction path is realized, which provides a more mature theoretical basis for optimizing the gene extraction path.

## 2 Literature review

It is not a novel thing to study biological genes to help people better understand and use biological genes. Xu L B discovered in his research on biological genes that biological rhythms are an important mechanism for organisms to receive external signals and regulate their own behavior. To this end, he summarized some core biological rhythms including biological clocks to assist biological breeding and human disease prevention and control (Xu et al., 2020). Ebigwai J K found that most of the existing protein research tools are only useful for the study of protein interaction PPI, so he proposed a new comprehensive PPI information extraction tool for support vector machine classifier, which is very important for protein gene research (Ebigwai et al., 2020). In order to better identify and predict the encoding of the genome, Abbas B proposed a new tool to rank, compare and identify the recurring properties in the hidden Markov model (HMM) (Abbas et al., 2019). Hasan M was opposed to synthetic gene editing and transgenic technologies that are widely used in food research and development. He hoped that the relevant departments can formulate more perfect biosafety regulations to reduce the risks related to food safety (Hasan et al., 2017). Do H pointed out that the mining technology of association rules can promote the study of different gene expression. To this end, she designed CPMiner, a data mining method for processing biological data, and dedicated to a unified framework for extracting gene expression using association rules (Do et al., 2020). Although these studies have carried out research on biological genes to a greater or lesser extent, they are all limited to a specific type or even a specific gene. However, this paper takes a unique approach to study the extraction path of biological genes, and provides a new research direction for the current gene research.

In recent years, with the spread of the concept of knowledge graph, more and more people have begun to conduct in-depth research on knowledge graph. Am A pointed out that the types of knowledge graphs are very broad, including public and private types. Knowledge graphs can also be obtained through the Internet, which requires statistical and linguistic methods (AlMarshad et al., 2021). Cai J L pointed out that a new multi-layer convolutional network model can be used in the model construction and connection prediction of large-scale knowledge graphs, which can build highly complex knowledge graphs based on its high parameter efficiency (Cai et al., 2019). Nuaima R H applied a new learning framework in the study of reasoning large-scale knowledge graphs (KG), which can construct knowledge graphs with continuous states in the KG vector space more accurately, diversely and efficiently (Nuaima et al., 2018). Shi J found that existing self-symptom detectors are far from meeting the needs of clinical decision support systems. Therefore, she studied an automated knowledge graph that can automatically learn diseases and conditions in electronic medical records to identify patients’ diseases (Shi et al., 2017). Lin Y Z found that the time limit is an invisible challenge to the mining and exploration of large-scale knowledge graphs. To this end, she proposed an online mining algorithm, which can greatly improve the speed and accuracy of mining knowledge graphs within a certain period of time (Lin et al., 2017a). Such a comprehensive knowledge map research provides a pioneering idea for the combination of knowledge map and biological gene extraction path in this paper. This paper looks forward to solving the problem of biological gene extraction path that has puzzled researchers for a long time by using the related theory of exponential graph and natural language processing.

## 3 Methods of biological gene extraction research based on knowledge graph and natural language processing

### 3.1 Knowledge graph and natural language processing

#### 3.1.1 Knowledge graph

**FIGURE 1:**
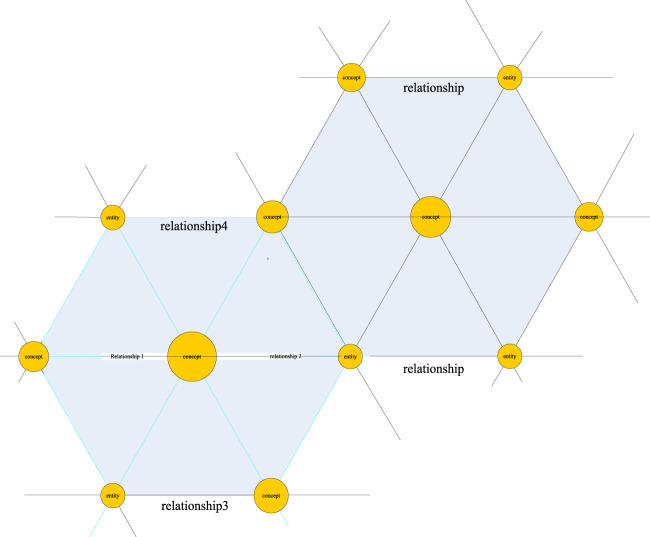
Knowledge graph network.

Knowledge graph is a semantic network proposed by Google in 2012 to describe the relationship between concepts in the real world in order to improve the search ability of its own engine (Paulheim and Cimiano, 2017). In fact, the essence of the knowledge graph is a conceptual network woven by related relationships between physical machines in the real world. As shown in Figure 1, on this network, nodes represent real objects or concepts, and the lines connecting each node are network edges and represent the correlation between them.

By combining the knowledge network with the Internet, it is possible to establish the relationship between things on the Internet with the help of the knowledge graph, and on this basis, to combine the all-encompassing information on the Internet to create new knowledge, which is a major breakthrough in artificial intelligence. The knowledge graph has been inseparable from artificial intelligence since it was proposed, and is widely used in search engines, intelligent question answering, and personalized recommendation (Lin et al., 2017b). The combination of the two can reduce the tediousness of manual search, and use Internet tools to visualize the searched knowledge.

**FIGURE 2:**
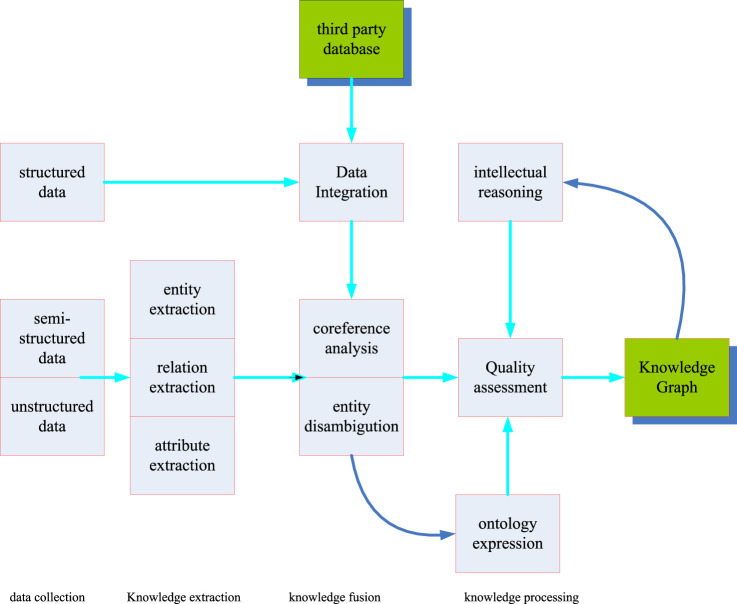
Construction process of the knowledge graph.

The construction process of the knowledge graph is based on the original data and use technical means to extract objective knowledge facts from the original data, then extracting knowledge elements from the knowledge facts and storing knowledge elements in a database. The construction of the knowledge graph hides the update of the knowledge graph (Zhang et al., 2017). Because the real world is constantly moving and changing, and new concepts are produced every moment, along with the demise of concepts, the construction of knowledge on this basis also means the renewal of knowledge. As shown in Figure 2, the construction of knowledge graph includes three stages, namely knowledge extraction, knowledge fusion and knowledge processing. Each knowledge construction is an iterative process of updating.

Among them, information extraction is the starting link of knowledge graph construction (Natthawut and Ryutaro, 2018). This link is mainly the process of extracting entities and the interrelationships of entity attributes from real entities. Through this link, a large number of discrete entities and their interrelationships can be achieved. Then, through the knowledge fusion link, the data obtained in the information extraction link is cleaned and classified. The redundant and repeated data are eliminated, and errors are eliminated to improve the hierarchy and logic of knowledge. Actually this coincides with the research on the biological gene extraction pathway studied in this paper. The research topic of this paper is to extract the core genes from the complex genome. The third link is the knowledge processing link. In this link, knowledge needs to be processed to obtain systematic and structured knowledge. In addition, a dynamic knowledge network needs to be finally obtained by constructing ontology, inferring knowledge and evaluating quality.

#### 3.1.2 Natural language processing

**FIGURE 3:**
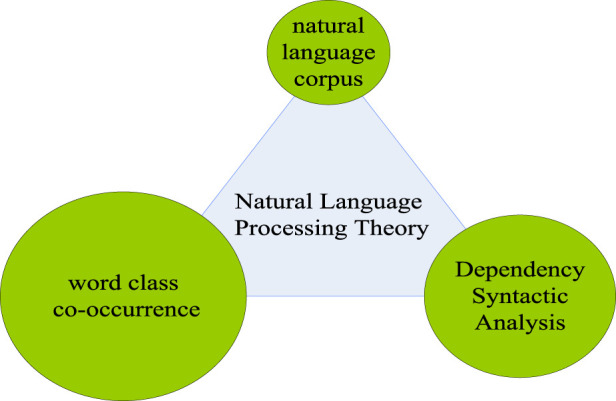
Natural language processing theory.

Natural language processing (NLP) is an important research tool for computers, especially in artificial intelligence. It mainly studies the means of communication between humans and computers, that is, natural language (Jia et al., 2017). In the information age, people can access information on the Internet more quickly because of natural language processing, and can also respond more quickly to changes in online life. The most typical example is the interception of e-mail spam. With the help of natural language processing, people can easily set up blocking words, so that the mailbox can block spam by locking key words to avoid harassment. The processing of natural language is inseparable from the natural language corpus. Natural language corpus is an indispensable foundation for processing natural language. Meanwhile, with the increasing development of natural language processing related work, the theory of natural language processing has gradually matured. As shown in Figure 3, the processing theory of natural language includes three parts: natural language corpus, dependency syntax analysis and part-of-speech co-occurrence (Zhu et al., 2017).

The study of natural language corpus comes from the interdisciplinary subject of corpus linguistics. Corpus linguistics is a hybrid science that combines linguistics, computer science and applied linguistics (Wang et al., 2018). Through the study of a large number of real language materials, this discipline summarizes some abstract language laws that are detached from specific languages and words, and applies these language laws to natural language processing, which plays an important role in improving the language learning ability of the machine. Corpus linguistics has been around for a hundred years since its inception, and so far corpora are still an integral part of natural language processing.

**FIGURE 4:**
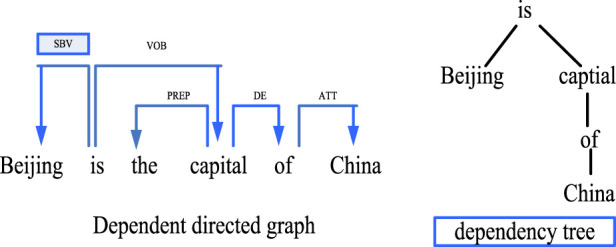
Process of dependency parsing.

Dependency parsing is an important part of natural language processing. Its working principle is mainly to reveal its intrinsic syntactic structure by analyzing the dependencies between various language components, and to assist the research by constructing a corresponding dependency tree. The workflow of dependency parsing is based on the existence of dependencies between different natural language units. Generally speaking, a dependency relationship consists of two parts, the core word and the modifier. Researchers can analyze the structure of sentences through dependency syntax analysis technology to determine the main components of sentences, so as to help machines better understand natural language and achieve more efficient human-computer interaction. As shown in Figure 4, the analysis process of dependency parsing is as follows:

Co-occurrence theory, literally, is a theory that studies the relationship of things that occur together (Tom et al., 2018). Generally speaking, the co-occurrence relationship is the most basic relationship in the entity relationship. In fact, the co-occurrence relationship refers to inferring the affinity between different entities by counting the number of co-occurrences between them in the same text, and this relationship is also called co-occurrence relationship in the field of natural language. Co-occurrence relationship can be divided into broad and narrow sense. Different from the limited scope of the co-occurrence relationship in the narrow sense, the generalized contribution relationship expands the scope of this relationship to time and space, and when analyzing this generalized co-occurrence relationship, it can further increase the prediction of the real world. For example, Walmart found that sales of egg tarts and flashlights can increase during the hurricane season, and the increase in sales and the arrival of a hurricane are co-occurrences in a broad sense. Based on this broad co-occurrence, Walmart would put together egg tarts and anti-hurricane supplies before the hurricane to increase sales.

### 3.2 Feature gene extraction based on knowledge graph and natural language processing

#### 3.2 1 Feature gene extraction

Feature gene extraction refers to finding out the genes that are not relevant for classification, extracting those representative genes. Then these genes are grouped into a subset for classification (Wong et al., 2018). There are two feature selection methods, namely feature selection and feature extraction. In a broad sense, feature extraction refers to a kind of mapping. The main methods are principal component method and partial least squares method. Feature selection only removes redundant genes and noise genes. Feature selection is difficult to achieve the optimal classification effect because the misclassification rate is too large, which is not considered in this paper.

#### 3.2 2 Scoring guidelines for gene expression data

In view of the high-dimensional and high-noise characteristics of gene expression, this paper believes that dimensionality reduction and denoising of gene expression are very necessary. Scoring guidelines are integral to this process. This paper mainly uses feature score criterion (FSC) and Fisher discriminant method. The scoring formulas are shown in Formulas 1, 2:

$$FSC\left(g_{i}\right)=\left|\frac{\eta_{i}^{+}-\eta_{i}^{-}}{\omega_{i}^{+}-\omega_{i}^{-}}\right|$$ (1)

$$FDR\left(g_{i}\right)=\frac{{\left(\eta_{i}^{+}-\eta_{i}^{-}\right)}^{2}}{{\left(\omega_{i}^{+}\right)}^{2}-{\left(\omega_{i}^{-}\right)}^{2}}$$ (2)

In Formula 1 and Formula 2, $\eta_{i}^{+}$ and $\eta_{i}^{-}$ represent the mean, while $\omega_{i}^{+}$ and $\omega_{i}^{-}$ represent the standard deviation.

#### 3.2.3 Feature gene extraction method

The purpose of eigengene extraction is to extract the most eigengenes from a group of genes, and the number of a group of eigengenes is extremely large in the experiment. Therefore, the selection of the extraction method of eigengenes is very important. The commonly used eigengene selection methods are as follows:

Filtering method: The filtering method refers to the selection according to the data of the information itself. This paper mainly uses the following two filtering methods, namely the signal-to-noise ratio and the t-statistic method. The signal-to-noise ratio is the simplest filtering method. The criterion for selecting feature vectors by using the signal-to-noise ratio method is the degree of difference in the vector attribute space in different categories of samples. Assuming that there is a gene g), the calculation formula of its signal-to-noise ratio $d\left(g\right)$ is as Formula 3:

$$d\left(g\right)=\left|\frac{\eta_{g}^{+}-\eta_{g}^{-}}{\omega_{g}^{+}-\omega_{g}^{-}}\right|$$ (3)

In Formula 3, $\eta_{g}^{+}$ and $\eta_{g}^{-}$ represent the mean, while $\omega_{g}^{+}$ and $\omega_{g}^{-}$ represent the standard deviation.

The t-statistic method refers to the use of the t-part theory to infer the probability of the occurrence of differences, and its calculation formula is shown in Formula 4:

$$t_{i}=\frac{{\bar{x}}_{i}-\bar{y_{i}}}{\sqrt{\frac{s_{1i}^{2}}{n_{1}}+\frac{s_{2i}^{2}}{n_{2}}}}$$ (4)

In Formula 4, ${\bar{x}}_{i}$ and $\bar{y_{i}}$ represent the mean, and $s_{1i}^{2}$ and $s_{2i}^{2}$ represent the variance. It can be seen that the larger the calculated t value, the greater the difference, that is, the greater the influence of gene i on the sample classification.

### 3.3 Path of feature gene extraction based on PCA

Gene expression data are characterized by small samples, high dimensionality, and high noise, and there are very few genes associated with diseases. The presence of a large number of redundant genes makes the expression difference between the diseased and normal samples small, and the contribution to the classification is also small. Therefore, it is necessary to use a certain gene microarray technology to propose genes from a large number of genes, and extract biological genes that truly meet the characteristics (Balsmeieri et al., 2018).

#### 3.3.1 Construction of gene expression data

Gene data covers the basic characteristics of genes and is a display of the physiological state of biological cells. The gene expression profile can be obtained by using gene chip technology. The expression profile data can be expressed as Formula 5:

$$X=\left(x_{ij}\right)=\left[\begin{array}{cccc} x_{11} & x_{12} & ... & x_{1j}\\ x_{21} & x_{22} & ... & x_{2j}\\ \vdots & \vdots & ... & \vdots \\ x_{i1} & x_{i2} & ... & x_{ij} \end{array}\right]$$ (5)

In Formula 5, $x_{ij}$ represents the j -th sample for the i -th gene.

#### 3.3.2 Elimination of irrelevant genes

There are tens of thousands of genes in a biological sample, and at least a few are needed for the experiment. Other unrelated genes can cause gene redundancy and interfere with the experiment. Therefore, this paper chooses a hybrid gene extraction method that combines the Bhattacharyya distance and the Gini index. The formula for calculating the Bhattacharyya distance of a gene is shown in Formula 6:

$$B_{d}=\frac{1}{8}\frac{{\left(\eta_{1}-\eta_{2}\right)}^{2}}{\left(\omega_{1}^{2}+\omega_{2}^{2}\right)}+\frac{1}{2}\ln\left(\frac{\omega_{1}^{2}+\omega_{2}^{2}}{2\omega_{1}\omega_{2}}\right)$$ (6)

Among them, $\eta_{1}$ and $\eta_{2}$ represent the mean of genes in the first and second samples respectively, while $\omega_{1}$ and $\omega_{2}$ represent the sample variance.

Gini index is an index commonly used in data mining to evaluate the goodness of classification nodes (Wi et al., 2017). Before calculating the Gini index, the original data should be discretized. The discrete formula is shown in Formula 7:

$$S_{ij}=\mathrm{Int}\left[20\times \frac{n_{ij}-\min\left(i\right)}{\max\left(i\right)-\min\left(i\right)}+0.5\right]$$ (7)

Among them, $\mathrm{Int}\left[\right]$ represents rounding, and $\max\left(i\right)$ and $\min\left(i\right)$ represent the maximum and minimum values of gene $g_{i}$ .

The discretized data is substituted into Formula 8:

$$Gini\left(k\right)=1-\overset{20}{\underset{j=0}{\sum }}{\left[p_{ij}\right]}^{2}$$ (8)

In Formula 8, $Gini\left(k\right)$ refers to the Gini index of *k*-type genes, and $p_{ij}$ refers to the relative rate of level *j* in category *k*.

The formula for calculating the Gini index of gene $g_{i}$ is as Formula 9:

$$Gini\left(g_{i}\right)=\overset{2}{\underset{k=1}{\sum }}\frac{n_{k}}{n}Gini\left(k\right),i\in \left[1,2000\right]$$ (9)

In Formula 9, *n* is the total number of samples and $n_{k}$ is the number of samples in the *k*-th class. Therefore, the smaller the Gini value of gene $g_{i}$ , the more taxonomic information this gene contains, and *vice versa*.

The Bhattacharyya distance and the Gini index are combined together, and these two indicators are integrated as a standard to measure the gene classification information, which avoids the defect of “signal-to-noise ratio” and other methods that do not generate variance in the differential expression of genes (Diamantopoulos et al., 2017).

#### 3.3.3 Selection of principal component feature

PCA refers to using the idea of dimensionality reduction to replace many original variables with new variables on the basis of keeping the principal components unchanged. These new variables can reflect most of the information of the original variables (Johnny et al., 2017; UzmaAl-Obeidat et al., 2022). The purpose of principal component analysis is to extract as few comprehensive variables with strong classification ability as possible as the representative of all variables, so as to retain the original variable information and reduce the number of variables. Assuming that there are *n* samples, and each sample contains *m* variables, then there is Formula 10:

$$X_{m\times n}=\left[\begin{array}{cccc} x_{11} & x_{12} & \cdots & x_{1m}\\ x_{21} & x_{22} & \ldots & x_{2m}\\ \vdots & \vdots & \vdots & \vdots \\ x_{n1} & x_{n2} & \ldots & x_{nm} \end{array}\right]$$ (10)

On this basis, the data is standardized to obtain Formula 11, Formula 12, Formula 13:

$$x_{ij}=\frac{x_{ij}-\bar{x_{j}}}{h_{j}}$$ (11)

$$\bar{x_{j}}=\frac{1}{n}\overset{1}{\underset{n=1}{\sum }}x_{ij}$$ (12)

$$h_{j}=\sqrt{\frac{1}{n-1}}{\overset{n}{\underset{i=1}{\sum }}\left(x_{ij}-\bar{x_{j}}\right)}^{2}$$ (13)

$\bar{x_{j}}$ means the mean of the j -th attribute, and $h_{j}$ means that the variance is normalized to have a mean of 0 and a standard deviation of 1 for each data.

Then the correlation coefficient matrix is obtained as Formula 14:

$$P=\left[\begin{array}{cccc} P_{11} & P_{12} & \cdots & P_{1t}\\ P_{21} & P_{22} & \ldots & P_{2t}\\ \vdots & \vdots & \vdots & \vdots \\ P_{m1} & P_{m2} & \ldots & P_{mt} \end{array}\right]$$ (14)

The correlation coefficient $p_{ij}$ represents the correlation between $x_{i}$ and $x_{j}$ , and the calculation formula is as Formula 15:

$$p_{ij}\frac{\overset{n}{\underset{k=1}{\sum }}\left(x_{ki}-\bar{x_{i}}\right)\left(x_{kj}-\bar{x_{j}}\right)}{\sqrt{\overset{n}{\underset{k=1}{\sum }}{\left(x_{ki}-\bar{x_{i}}\right)}^{2}\overset{n}{\underset{k=1}{\sum }}{\left(x_{kj}-\bar{x_{j}}\right)}^{2}}},\;i,j=1,2,...,m$$ (15)

Solving the characteristic formula according to the correlation coefficient matrix *P*, there is Formula 16:

$$\left|\gamma I-P\right|=0$$ (16)

The characteristic formula is solved to get different eigenvalues. Sorting these eigenvalues yields Formula 17:

$$\gamma_{1}\geq \gamma_{2}\geq \cdots \geq \gamma_{m}\geq 0$$ (17)

Then the standard orthonormal vector is calculated by the existing eigenvalues to obtain Formula 18:

$$e_{i}={\left(e_{1i},e_{2i},..,e_{mi}\right)}^{T}$$ (18)

On this basis, the analysis of different principal component contribution rates obtains Formula 19:

$$\lambda_{i}=\frac{\gamma_{i}}{\overset{m}{\underset{k=1}{\sum }}\gamma_{k}},i$$ (19)

After calculating the contribution rate of each principal component, the cumulative sum is obtained to obtain the cumulative contribution rate as shown in Formula 20:

$$\lambda_{\left(i\right)}=\frac{\overset{i}{\underset{k=1}{\sum }}\gamma_{k}}{\overset{m}{\underset{k=1}{\sum }}\gamma_{k}},i=1,2,...,m$$ (20)

Finally, the gene contribution rate Formula 21 can be found:

$$g_{j}=\frac{\overset{t}{\underset{i=1}{\sum }}\left|d_{ij}\right|}{\overset{t}{\underset{i=1}{\sum }}\overset{m}{\underset{k=1}{\sum }}\left|d_{ik}\right|},j=1,2,..,m$$ (21)

## 4 Experiment and results of feature gene extraction based on PCA

### 4.1 Experimental data acquisition and processing

With the fast-paced work and life, the living pressure of modern women has greatly increased. Staying up late and insomnia are quite common. In such a stressful life rhythm, breast cancer, the killer disease of women’s health, has quietly appeared in more and more women. In order to alleviate this situation, this paper took breast cancer as the research object, and analyzed the characteristic gene extraction pathway of breast cancer, hoping to enrich the breast cancer gene extraction pathway and contribute to the control and prevention of breast cancer.

In order to verify the feature gene extraction path proposed in this paper, several common disease gene datasets were collected in this paper, as shown in Table 1:

**TABLE 1:** Sample gene datasets.

Datasets	Category	Sample amount	Gene amount
Leukemia data set	ALLI.ALLB.AWL	46	6844
Polio data set	4	22	3629
Breast cancer data set	3	50	2000
Colon cancer data set	ALL.AML	82	96548

As shown in Table 1, the total number of samples in the selected four diseases was 200 cases. It can be seen that the number of samples of breast cancer genes was large, but the total number of genes was relatively small. The former satisfied the quantitative requirements of the experiment, and the latter represented the less complex number of genes, which was convenient for screening. Even though all four of these diseases were common, the choice of breast cancer genes was more beneficial than other options.

Through the investigation, it can be seen that among the 50 samples in this breast cancer data set, 28 were diseased genes, and the remaining 22 were normal samples. In this regard, 30 samples were used as the training set, and the remaining 20 samples were used as the test set. The description of the samples is shown in Table 2:

**TABLE 2:** Basic description of sample data.

Breast cancer sample data division	Lesion sample	Normal sample
Sample size	28	22
Training set	20	10
Test set	8	12

### 4.2 Experimental process and results

#### 4.2.1 Elimination of irrelevant genes

Based on the characteristics of sample genes with small number of samples and many redundant genes, although there were 2000 genes in 20 samples, there were still a large number of genes that were not related to the content of the experiment. Therefore, this paper combined the two methods of Bhattacharyya distance and Gini index to measure to promote the standardization of gene classification. This method avoided the defect that methods such as “signal-to-noise ratio” did not express differential gene expression due to variance. On this basis, this paper focused on the former and the latter as a supplement, so that the priority and secondary could better extract public genes.

**FIGURE 5:**
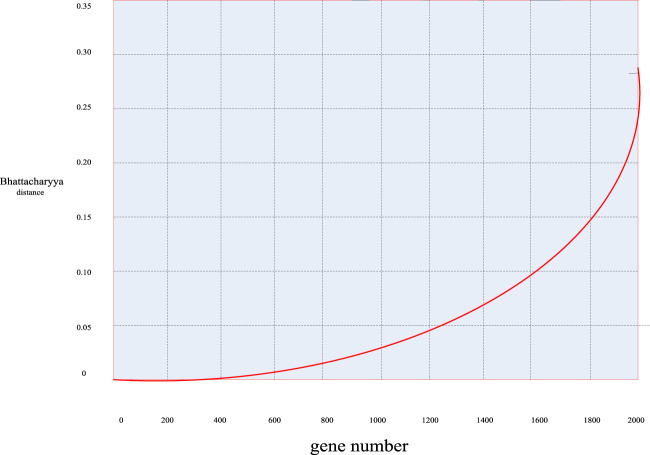
Bhattacharyya distance distribution curve of breast cancer genes.

**FIGURE 6:**
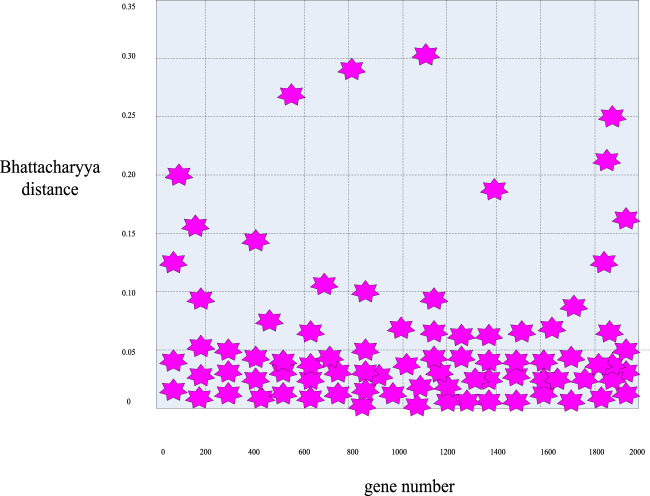
Scatter plot of the distribution of Bhattacharyya distance for breast cancer genes.

First, according to the distance calculation formulas mentioned above, the Bhattacharyya distance value of these 2000 sample genes was calculated to obtain the distribution curve diagram of the Bhattacharyya distance of these genes in Figure 5 and the distribution scatter diagram in Figure 6.

First, it can be seen from Figures 5, 6 that in general, only a small number of genes have high information content, while most of the genes have a Bhattacharyya distance index between 0 and 0.05. This indicated that the genes within this range had no significant difference in mean and variance between normal samples and cancer samples, so it can be easily deleted as an irrelevant gene. Considering the validity and accuracy of the experiment, in this experiment, the Bhattacharyya distance index of these 2000 genes was sorted from small to large, and the corresponding genes ranked in the bottom 200 were selected as further experimental objects, eliminating the other 1800 irrelevant genes.

**FIGURE 7:**
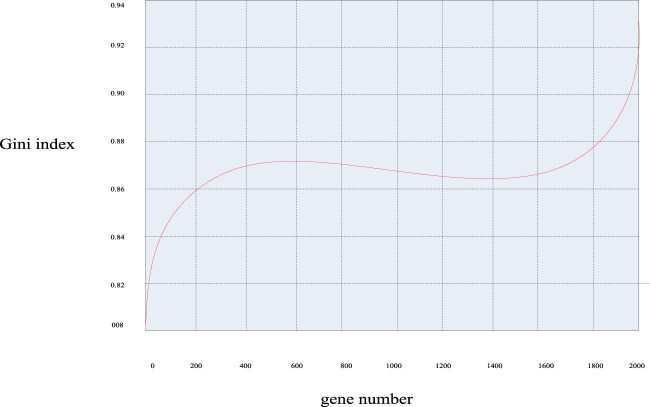
Gini index distribution curve of breast cancer genes.

**FIGURE 8:**
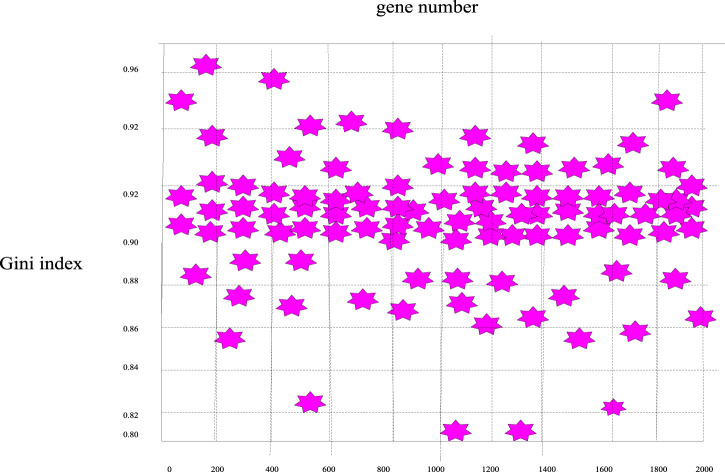
Scatter plot of the distribution of Gini index distribution of breast cancer genes.

Similarly, according to the calculation formula of the Gini index mentioned above, the Gini index values of these 2000 genes were calculated, and the Gini index distribution curve diagram of the sample genes can be obtained in Figure 7 and the distribution scatter diagram in Figure 8.

It can be seen from Figures 7, 8 that the Gini index values of these 2000 sample genes were mostly between 0.88 and 0.92. In fact, the smaller the Gini index of a gene, the more equal the data expression, and the greater the amount of classification information of the gene. Since the experimental design of this paper was mainly based on the Bhattacharyya distance ranking, supplemented by the Gini index ranking, the same sorting method was used to eliminate irrelevant genes, and 200 spare genes were extracted.

After selecting 200 genes for each of the Bhattacharyya distance index and the Gini index as experimental subjects, among these 400 genes, the common genes were found whose duplicates were regarded as both. It could be determined that the amount of taxonomic information in these public genes far exceeded that of the excluded irrelevant genes. In the end, 49 common genes were obtained.

#### 4.2.2 Extraction of characteristic genes

The dimensionality reduction idea of PCA was used to select the features of the 49 common genes finally selected, and the feature genes could be selected by the contribution rate of each gene in the finally selected principal components.

The eigenvalue, contribution rate and feature contribution rate of each principal component obtained by PCA of the 49 public genes are shown in Table 3:

**TABLE 3:** List of principal component characteristic data of public genes.

Main ingredient	Eigenvalues	Contribution rate (%)	Feature contribution rate (%)
A	28.174	39.658	39.658
B	10.742	18.649	49.165
C	9.645	12.359	57.761
D	8.742	8.542	61.952
E	4.568	3.406	71.962
F	2.691	2.864	77.294
G	1.669	2.173	79.429
H	1.485	1.749	81.263
I	1.293	1.599	83.648

It can be seen from Table 3 that the eigenvalues of the first six principal components were all greater than 2, and the eigenvalues corresponding to the seventh principal component were all less than 2. The cumulative contribution rate of the first six principal components has reached 85.478%. Therefore, only the first six principal components could be further analyzed.

**FIGURE 9:**
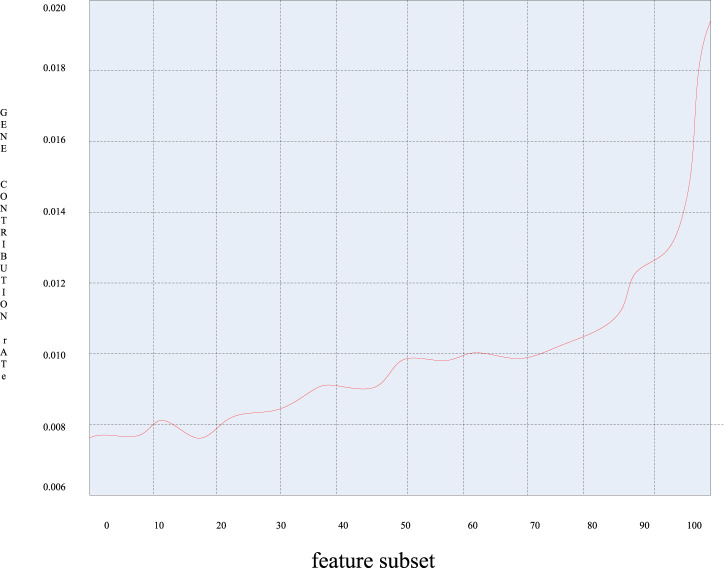
Graph of the contribution rate of each principal component.

When further analyzing the first six principal components, the contribution rate of each principal component has been calculated, and Figure 9 was obtained by arranging the contribution rate of each principal component in ascending order:

It can be seen from Figure 9 that the contribution rate of these 49 genes varied greatly, and the gene contribution rate varied greatly between 0.010 and 0.018. That is to say, those genes with a large gene contribution rate contain more classification information of the sample and contribute more to the classification. It can be seen that these genes contain a great amount of information, and the corresponding sample genes can be extracted as characteristic genes.

#### 4.2.3 Classification inspection

On the basis of extracting the eigengenes above, this paper used the support vector machine (SVM) radial basis kernel function to detect the extracted eigengenes. The results are shown in Table 4:

**TABLE 4:** Detection results of signature genes.

Number of genes	Classification accuracy (%)	Number of misjudgments
6	78.82	7
10	84.62	5
5	90.31	4
16	67.74	5
20	88.52	4

It can be seen from Table 4 that when the number of selected eigengenes was 5, the recognition rate of genes reached a maximum of 90.31%. Therefore, it can be determined that only 5 genes in the 2000 sample genes finally contained rich information and meet the experimental requirements. The 5 most useful genes were extracted from 2000 genes, and the rate of removing redundant genes reached 99.75%, which just proved that the characteristic gene extraction path designed in this paper has a good classification effect on breast cancer genes.

## 5 Conclusion

This paper studied the extraction path of biological genes related to knowledge graph and natural language processing. The main significance of this research was to provide more abundant channels for gene extraction in modern medicine. In the past biological gene extraction research, most people were limited to the activity of the extracted genes, but ignored how to accurately extract the characteristic genes from the source to better meet the experimental needs, which is also the starting point of this study. In this regard, the sample genes of the research object were standardized by two screening criteria, the Bhattacharyya distance index and the Gini index. Then the PCA method was used to further extract the eigengenes, and finally the eigengenes were verified. It was proved that the redundant gene removal rate of the characteristic gene extraction method in this paper reached 99.75%, which provides a certain reference value for modern biological research.

## Data Availability

The original contributions presented in the study are included in the article/supplementary material, further inquiries can be directed to the corresponding author.

## References

[B1] Abbas B. Tjolli I. Dailami M. Munarti (2019). Phylogenetic of sago palm (Metroxylon sagu) and others monocotyledon based on mitochondrial nad2 gene markers. Biodiversitas J. Biol. Divers. 20 (8), 2249–2256. 10.13057/biodiv/d200820

[B2] AlMarshad L. K. AlJobair A. M. Al-Anazi M. R. Bohol M. F. F. Wyne A. H. Al-Qahtani A. A. (2021). Association of polymorphisms in genes involved in enamel formation, taste preference and immune response with early childhood caries in Saudi pre-school children. Saudi J. Biol. Sci. 28 (4), 2388–2395. 10.1016/j.sjbs.2021.01.036 33911954 PMC8071886

[B3] Balsmeieri B. Assaf M. Chesebro T. Fierro G. Johnson K. Johnson S. (2018). Machine learning and natural language processing on the patent corpus: Data, tools, and new measures. J. Econ. Manag. Strategy 27 (3), 535–553. 10.1111/jems.12259

[B4] Cai J. L. Yan Y. F. Feng G. W. Jing S. (2019). Dynamic change in the gene expression profile of rat benign prostate hyperplasia tissue after complete denervation. Zhonghua nan ke xue = Natl. J. Androl. 25 (11), 971–977. 32233229

[B5] Diamantopoulos T. Roth M. Symeonidis A. Klein E. (2017). Software requirements as an application domain for natural language processing. Lang. Resour. Eval. 51 (2), 495–524. 10.1007/s10579-017-9381-z

[B6] Do H. Thu T. Tran N. (2020). Indigenous Lien Minh chicken of Vietnam: Phenotypic characteristics and single nucleotide polymorphisms of GH, IGFBP and PIT candidate genes related to growth traits. Biodiversitas J. Biol. Divers. 21 (11), 5344–5352.

[B7] Ebigwai J. K. Ferdinand A. Ubi G. M. (2020). Resolving taxonomic ambiguity between two morphological similar plant taxa using maturase K gene analysis. J. Biol. Sci. 20 (1), 13–21. 10.3923/jbs.2020.13.21

[B8] Hasan M. Siddique M. A. Hossain M. A. Rahman M. S. (2017). 16S rRNA gene sequence based identification of Vibrio spp. in shrimp and tilapia hatcheries of Bangladesh. Dhaka Univ. J. Biol. Sci. 26 (1), 45–58. 10.3329/dujbs.v26i1.46349

[B9] Jia Y. Wang Y. Jin X. Lin H. Cheng X. (2017). Knowledge graph embedding: A locally and temporally adaptive translation-based approach. ACM Trans. Web 12 (2), 1–33. 10.1145/3132733

[B10] Johnny D. Velupillai S. George G. Holden R. Kikoler M. Dean H. (2017). Detection of suicidality in adolescents with autism spectrum disorders: Developing a Natural Language Processing approach for use in electronic health records. AMIA Symp. 2017, 641–649. PMC5977628 29854129

[B11] Lin Y. Z. Ou D. L. Chang H. Y. Lin W. Y. Hsu C. Chang P. L. (2017). Simultaneous visualization of the subfemtomolar expression of microRNA and microRNA target gene using HILO microscopy. Chem. Sci. 8 (9), 6670–6678. 10.1039/c7sc02701j 28989695 PMC5625256

[B12] Lin Z. Q. Xie B. Zou Y. Z. Zhao J. F. Li X. D. Wei J. (2017). Intelligent development environment and software knowledge graph. J. Comput. Sci. Technol. 32 (002), 242–249. 10.1007/s11390-017-1718-y

[B13] Natthawut K. Ryutaro I. (2018). An automatic knowledge graph creation framework from Natural Language text. Ieice Trans. Inf. Syst. 101 (1), 90–98. 10.1587/transinf.2017swp0006

[B14] Nuaima R. H. Roeb J. Hallmann J. Daub M. Otte S. Heuer H. (2018). Effector gene vap1 based DGGE fingerprinting to assess variation within and among *Heterodera schachtii* populations. J. nematology 50 (4), 517–528. 10.21307/jofnem-2018-055 PMC6909312 31094153

[B15] Paulheim H. Cimiano P. (2017). Knowledge graph refinement: A survey of approaches and evaluation methods. Semantic Web 8 (3), 489–508. 10.3233/sw-160218

[B16] Shi J. Li W. Gao Y. Wang B. Li Y. Song Z. (2017). Enhanced rutin accumulation in tobacco leaves by overexpressing the NtFLS2 gene. Bioence Biotechnol. Biochem. 81 (9), 1721–1725. 10.1080/09168451.2017.1353401 28715245

[B17] Tom Y. Devamanyu H. Soujanya P. Cambria E. (2018). Recent trends in deep learning based Natural Language Processing. IEEE Comput. Intell. Mag. 13 (3), 55–75. 10.1109/mci.2018.2840738

[B18] UzmaAl-Obeidat F. Tubaishat A. Shah B. Halim Z. (2022). Gene encoder: A feature selection technique through unsupervised deep learning-based clustering for large gene expression data. Neural Comput. Applic 34, 8309–8331. 10.1007/s00521-020-05101-4

[B19] Wang C. Ma X. Chen J. (2018). Information extraction and knowledge graph construction from geoscience literature. Comput. Geosciences 112, 112–120. 10.1016/j.cageo.2017.12.007

[B20] Wi C. L. Sohn S. Rolfes M. C. Seabright A. Ryu E. Voge G. (2017). Application of a Natural Language Processing algorithm to asthma ascertainment: An automated chart review. Am. J. Respir. Crit. Care Med. 196 (4), 430–437. 10.1164/rccm.201610-2006OC 28375665 PMC5564673

[B21] Wong A. Plasek J. M. Montecalvo S. P. Zhou L. (2018). Natural Language processing and its implications for the future of medication safety: A narrative review of recent advances and challenges. Pharmacother. J. Hum. Pharmacol. Drug Ther. 38 (8), 822–841. 10.1002/phar.2151 29884988

[B22] Xu L. B. Zhao Z. G. Xu S. F. Zhang X. X. Liu T. Jing C. Y. (2020). The landscape of gene mutations and clinical significance of tumor mutation burden in patients with soft tissue sarcoma who underwent surgical resection and received conventional adjuvant therapy. Int. J. Biol. Markers 35 (3), 14–22. 10.1177/1724600820925095 32520634

[B23] Zhang C. Miao Z. Xiao H. Hu Z. Ji Y. (2017). Knowledge graph embedding for hyper-relational data. Tsinghua Sci. Technol. 22 (2), 185–197. 10.23919/tst.2017.7889640

[B24] Zhu Y. Zhou W. Xu Y. Liu J. Tan Y. (2017). Intelligent learning for knowledge graph towards geological data. Sci. Program. 2017 (1), 1–13. 10.1155/2017/5072427

